# Electroacoustic Verification Comparison of AirPods Pro 2nd and 3rd Generations and Traditional Hearing Aids

**DOI:** 10.3390/audiolres16020055

**Published:** 2026-04-09

**Authors:** Seeon Kim, Linda Thibodeau

**Affiliations:** 1 Callier Center for Communication Disorders, Richardson, TX 75080, USA; 2 Department of Speech, Language, and Hearing, University of Texas at Dallas, Richardson, TX 75080, USA; thib@utdallas.edu

**Keywords:** hearing aids, AirPods Pro, hearing technology

## Abstract

Background: The recent U.S. Food and Drug Administration authorization of AirPods Pro as over-the-counter hearing aids (HAs) has increased interest in consumer devices as potential alternatives to traditional amplification; however, their electroacoustic performance relative to clinically fitted HAs remains unclear. The purpose of this study was to compare the electroacoustic characteristics and real-ear measures of AirPods Pro 2nd generation (APP2), AirPods Pro 3rd generation (APP3), and a traditional receiver-in-the-canal HA across mild flat, mild-to-moderate sloping, and moderate flat hearing loss configurations. Methods: Outcome measures included 2cc coupler output curves, saturation sound pressure level for a 90 dB input (SSPL90), real-ear speech mapping, maximum power output (MPO), and real-ear-to-coupler differences. Results: Coupler-based electroacoustic measures showed that APP2 and APP3 produced output comparable to the traditional HA (within 7 dB). SSPL90 outputs were similar for APP2 and APP3, whereas the HA demonstrated profile-dependent increases. In contrast, real-ear measurements demonstrated that both APP2 and APP3 consistently produced less output relative to the HA that was fitted to NAL-NL2 targets, with the largest deviations observed for moderate hearing loss and at higher frequencies (up to 14 dB). Across all configurations, MPO was consistently highest for the HA, with both AirPods devices exhibiting reduced maximum output, especially in speech-critical frequency regions. Real-ear-to-coupler difference findings indicated reduced acoustic coupling for APP3 relative to APP2 and the HA, contributing to reduced in-ear amplification despite comparable coupler outputs. Conclusions: While AirPods Pro may offer benefit for mild hearing loss or moderate high-frequency hearing loss, they do not provide output comparable to prescriptively fitted HAs. These findings underscore the continued importance of clinical verification and prescription-based fitting of hearing assistive technology for achieving appropriate audibility across hearing loss configurations.

## 1. Introduction

According to Statista’s Global Consumer Survey, AirPods Pro dominated the U.S. headphone market, holding a 34.4% share [1]. Apple had sold over 75 million units of AirPods by the end of 2024, resulting in a user base exceeding 100 million worldwide [2]. In September 2024, the U.S. Food and Drug Administration (FDA) authorized AirPods Pro (2nd and 3rd generations) for use as an over-the-counter (OTC) hearing aid (HA) for individuals with perceived mild-to-moderate hearing loss, when configured to meet the user’s hearing needs [3]. This decision may have an impact on the underutilization of HAs by many individuals who cannot afford traditional clinical audiological services.

According to National Institute on Deafness and Other Communication Disorders nearly one-quarter of older adults in the United States experience hearing loss, predominantly due to age-related factors [4]. However, the adoption rate of HAs remains low, with only 38.4% of the eligible population using them in 2022 [5], possibly due to concerns about cost and cosmetic appearance [6,7,8,9,10]. AirPods Pro may offer a viable alternative for individuals with mild-to-moderate hearing loss given their relatively low cost, accessibility, and user-friendly design.

Apple released the AirPods Pro 2nd generation (APP2) in September 2022 and the AirPods Pro 3rd generation (APP3) in September 2025 [11]. APP3 offers five ear tip size options (XXS, XS, S, M, L), while APP2 provides four options (XS, S, M, L). APP3 provides up to 8 h of listening time per charge, compared to 6 h for APP2. The APP3 charging case does not include a Bluetooth pairing button on the back; instead, it offers touch-based Bluetooth pairing, whereas APP2 includes a physical pairing button. APP3 features larger microphone ports relative to APP2 and incorporates a heart rate sensor, which is not present in APP2. In terms of durability, APP2 is rated IP54 for dust, sweat, and water resistance, whereas APP3 is rated IP57. Apple reports that APP3 provides up to two times greater active noise cancelation compared to APP2.

APP2 and APP3 offer three listening modes, Transparency, Adaptive, and Noise Cancelation, which are accessible through the device settings or touch controls. Among these, Transparency mode supports HA like-amplification fitted to an individual’s hearing thresholds configured through the Apple Health application. Users can access the Hearing section in the Apple Health app to input hearing thresholds using results from an audiological evaluation or by completing an in-app hearing test administered via AirPods Pro. For prior test results, users may either scan an audiogram using the device camera or manually enter air- and bone-conduction thresholds for each test frequency. Alternatively, the in-app hearing test requires the user to complete the assessment in a quiet environment. The test automatically pauses when environmental noise increases and resumes when the environment becomes quiet again. The procedure includes an ear tip fit check, followed by pure-tone presentation using a three-beep sequence. Users are instructed to tap the screen when a tone is detected, with testing conducted sequentially for the left and right ears. The result provides an audiogram with threshold estimates across frequencies ranging from 250 to 8000 Hz, comparable to those obtained in a clinical audiological evaluation. Upon completion of the hearing test or entry of audiometric test results, users are prompted to turn on their hearing aid, which activates frequency-specific amplification tailored to the individual’s hearing profile. As required by the FDA for OTC HAs, AirPods Pro must comply with the American National Standards Institute/Consumer Technology Association (ANSI/CTA-2051) [12].

While AirPods Pro’s output is determined by audiometric thresholds manually entered by the user, traditional HAs, in contrast, provide extensive fine-fitting capabilities. For example, clinicians can select among prescriptive fitting formulas such as DSL, NAL-NL, or manufacturer-specific algorithms. DSL may prescribe higher gain, particularly in the high frequencies [13], and is more commonly used in pediatric fittings, whereas NAL-NL2 is widely used in adults to optimize speech intelligibility while maintaining overall loudness comfort [14]. HA fitting software (e.g., Phonak Target, Oticon Genie2, and Starkey InspireX) allows for detailed adjustment of frequency-specific gain across input levels (soft, moderate, and loud) as well as maximum power output (MPO). In addition, amplification can be adapted to different listening environments. For instance, Phonak Target provides Automatic Programs that can be adjusted for calm situations, speech in noise, comfort in noise, and music.

Previous research for AirPods Pro has focused on the evaluation of electroacoustic characteristics. The maximum output pressure level at 90 dB SPL input was reported to be 122–123 dB SPL for the AirPods 2nd generation, compared to 110 dB SPL for the AirPods Pro 1st generation (APP1) [15]. Real-ear insertion gain was assessed in typical-hearing participants with the Conversation Boost feature enabled on APP1 [16]. The findings indicated that AirPods Pro provided greater amplification in the 0.4–3 kHz frequency range but less amplification in the high-frequency range (5–8 kHz) relative to the NAL-NL2 prescription targets. Real-ear-measurements were also conducted in participants with bilateral hearing thresholds between 25 and 55 dB HL [17]. With the Headphone Accommodation feature enabled on APP1, the average difference between the target and measured responses were as great as 12.25 dB at 6 kHz, while differences were below 10 dB for frequencies between 0.25 and 4 kHz. Finally, APP1 was evaluated using customized settings corresponding to different degrees of hearing loss (flat 0-, 30, and 60 dB HL) [18]. Electroacoustic testing demonstrated increased output with increasing degrees of hearing loss; however, speech mapping conducted with Knowles Electronics Manikin for Acoustic Research suggested that the output may not have been sufficient for the 30 and 60 dB HL conditions.

Despite these findings, the electroacoustic characteristics of the newly FDA-approved amplification feature (Transparency mode available in APP2 and APP3) have not yet been evaluated across varying degrees and configurations of hearing loss. Verification of both generations of AirPods Pro as an OTC hearing device is essential to determine its acoustic performance. As AirPods Pro begins to be accepted as an alternative to conventional amplification, it is necessary to determine how closely their outputs approximate those of traditional HAs, which can be verified using the clinical standards for meeting prescriptive targets and providing frequency-specific gain across diverse hearing loss profiles. Given there are two generations of AirPods Pro that can be used as an OTC hearing device, both were included in this study. The purpose of this study is to provide comprehensive electroacoustic comparison of the performance of APP2, APP3, and traditional HAs across three configurations of hearing loss.

## 2. Materials and Methods

### 2.1. Devices

One right APP2 and APP3 were set to Transparency mode, and an iPhone 15 Pro (iOS 18) was set to maximum volume. The same ear tip (XS-sized) was used for both APP2 and APP3. The Transparency mode of APP2 and APP3 is activated only when the device is in contact with human skin; therefore, Ten20 Conductive Neurodiagnostic Electrode Paste was applied to the skin-detection sensor to maintain continuous activation throughout testing. Other features such as Media Assist were not enabled. Audiograms were manually entered in the Hearing Test Result section of the Apple Health App on the iPhone. The amplification provided by the AirPods Pro was determined by their proprietary algorithm rather than by using widely accepted fitting formula provided by most HA manufacturers.

One right Phonak Sphere 90 receiver-in-the-canal HA (Phonak, Sonova Group, Stäfa, Switzerland) was used to represent a traditional HA comparator for APP2 and APP3. A closed dome and medium-power receiver were used, which is suggested for those who have mild-to-moderate hearing loss and to allow comparison with AirPods Pro, which uses non-customized ear tips. Audiograms were entered into the Phonak Target 9.1 software, and the HA was programmed according to the NAL-NL2 prescriptive fitting targets for the three hearing loss configurations. All automatic programs and advanced features were disabled in AutoSense OS 4.0—including Speech Enhancer, SoundRelax, NoiseBlock, Soft Noise Reduction, WindBlock, and WhistleBlock.

### 2.2. Hearing Loss Configurations

To verify amplification, three audiograms were selected: one representing flat, mild hearing loss; another representing flat, moderate hearing loss; and a third representing mild-to-moderate high-frequency sloping hearing loss (Table 1). The sloping loss was selected to explore a common hearing loss that may occur with aging and likely be noticeable by the user [19].

### 2.3. Measurement Protocols

#### 2.3.1. Part 1: Electroacoustic Analyses

Electroacoustic analyses were conducted using the FONIX 8000 (Frye Electronics, Beaverton, OR, USA), interfaced with a Dell computer (Windows 10; Dell Inc., Round Rock, TX, USA) operating the FONIX WinCHAP HA software (Version 3.00). APP2, APP3, and HA were measured using an HA-1, 2cc coupler in the Frye test box when attached to the coupler in the same manner as a custom aid would be using fun-tak. Output was measured for the three hearing loss configurations under two conditions. The first condition was comparing output for a 65 dB SPL speech-weighted composite signal when it was randomly interrupted versus being played continuously to simulate speech and noise, respectively. The output curves for these signals were compared in order to examine potential noise reduction (NR) that may occur for the continuous signal. The second condition measured saturation sound pressure level for a 90 dB SPL pure-tone swept input (SSPL90).

Because AirPods Pro do not have manufacturer-specified guidelines for test box placement, the effect of placement was evaluated for APP3. With the AirPods Pro attached to the HA-1 coupler by surrounding the ear tip with fun-tak, four device positions (rotating the coupling 45 degrees) were evaluated and resulted in less than 1 dB variation across three measurement days. The vertical position (AirPods Pro microphone facing the test box speaker) was used for all electroacoustic measures.

#### 2.3.2. Part 2: Speech Mapping Real-Ear Measurement

Speech mapping real-ear-measurements were conducted using the AudioScan Verifit 2 (version 4.26.3) on the author’s right ear (female, 33 years). The Acoustic Seal Test (or Ear Tip Fit test) was passed. Output was measured for the three hearing loss configurations in two real-ear conditions: (1) output using the standard female speech signal at an average level of 65 dB SPL; (2) output using pure-tone swept at 85 dB SPL to determine MPO. Unlike in a clinical setting where the goal would be matching targets for a prescriptive fitting, the focus here was on comparing the outputs for the HA to the two generations of AirPods Pro devices. Therefore, results will be discussed with reference to the HA output rather than a prescriptive target.

#### 2.3.3. Part 3: Real-Ear-to-Coupler Difference

Real-ear-to-coupler difference (RECD) represents the difference between the coupler response and the real-ear response and is commonly used to account for individual ear canal acoustics. The output of the AirPods Pro or the RIC HA was measured using the HA-1 2cc coupler from the AudioScan Verifit 2 test box with the standard female speech signal presented at 65 dB SPL. This coupler output curve was subtracted from the real-ear response which was obtained with the probe tube inserted in the ear canal while wearing each device for the same input level as was used for the coupler measures. The difference between the two curves is the RECD.

Because this study was focused on a descriptive electroacoustic approach with a single subject, no inferential statistical analyses were performed.

## 3. Results

### 3.1. Part 1: Electroacoustic Analyses

**Figure 1:**
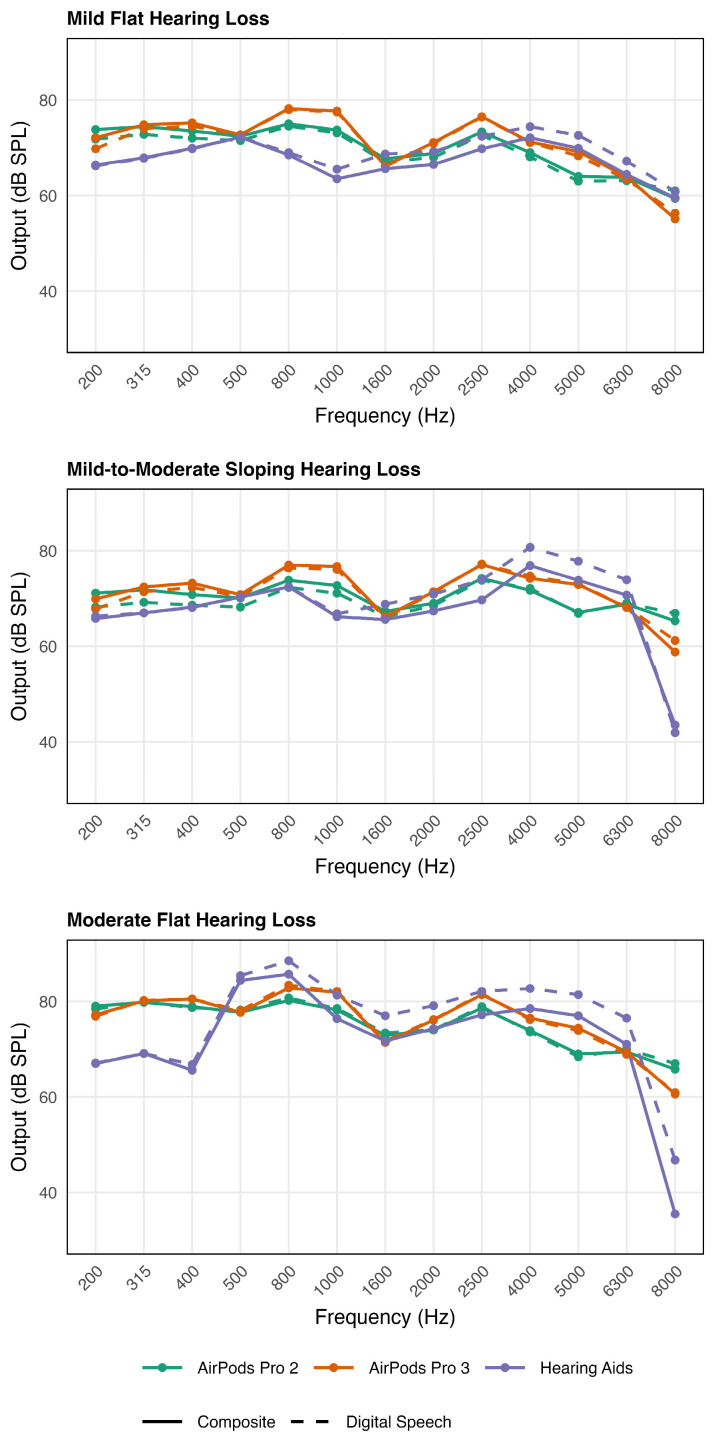
Output for 65 dB SPL input as a function of frequency for APP2, APP3, and HA under three hearing loss configurations: mild flat (**top**), moderate flat (**middle**), and mild-to-moderate (**bottom**) hearing loss. The solid lines represent the output for the composite signal when it was continuous versus the dashed lines that represent the output for the interrupted, i.e., digital, signal.

**Figure 2:**
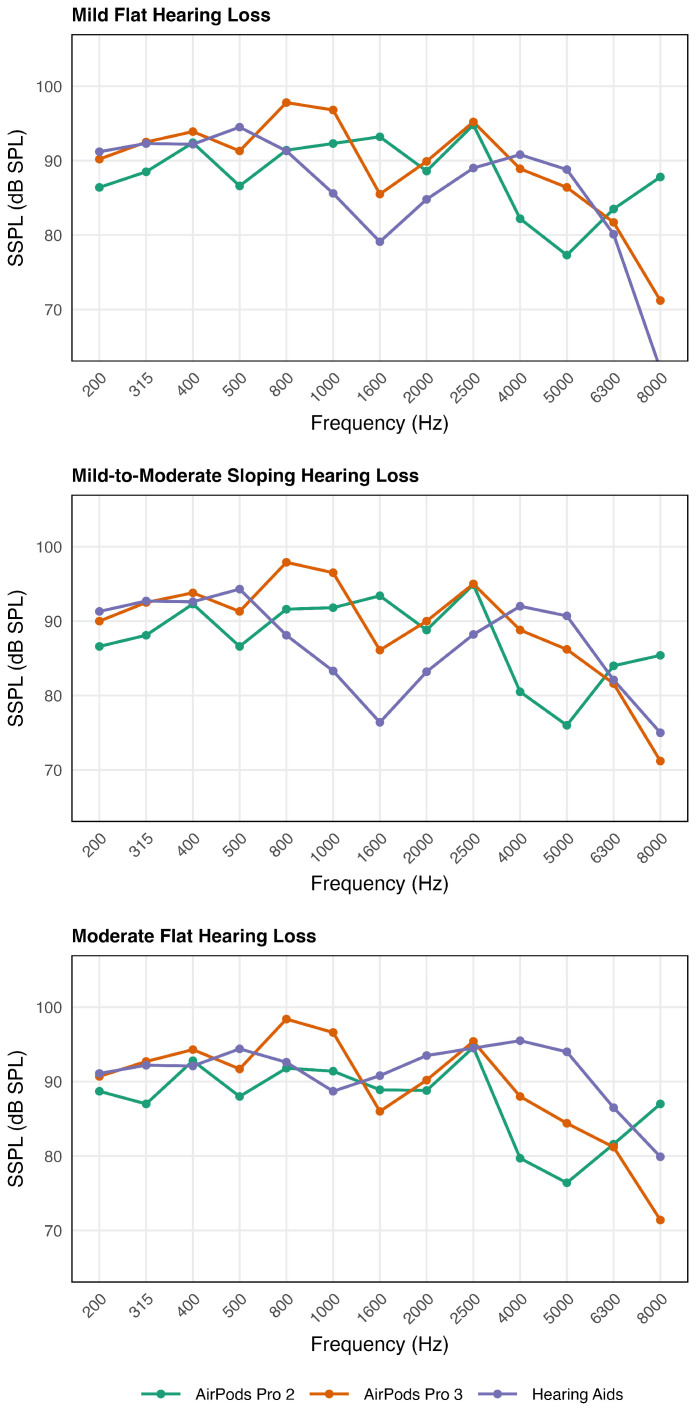
SSPL 90 (saturation sound pressure level for a 90 dB input) as a function of frequency for APP2, APP3, and HA under three hearing loss configurations: mild flat (**top**), moderate flat (**middle**), and mild-to-moderate (**bottom**) hearing loss.

The results are shown in Figure 1 and Figure 2 for the two electroacoustic measurements. There are three panels which represent the three hearing loss configurations.

#### 3.1.1. Condition (1) Output for 65 dB SPL Composite Signal (See Figure 1)

- Comparison across hearing loss conditions for digital speech
  1. Mild Flat Hearing Loss. For the digital speech signal, both APP2 and APP3 provided greater output in the low frequencies (200–1600 Hz), exceeding HA output by 0.5–12 dB and comparable output in the mid frequencies (1600–2500 Hz). In contrast, both APP2 and APP3 provided less amplification than the HA in the high frequencies (4000–8000 Hz), by 3.3–4.5 dB.
  2. Mild-to-Moderate Sloping Hearing Loss. For the digital speech signal, APP2 and APP3 generally provided greater output than the HA across the low-to-mid frequency ranges (200–1000 Hz), exceeding HA output by 0.1–9.3 dB. Similarly to the mild HL condition, both APP2 and APP3 provided less output than the HA in the high-frequency range (4000–6300 Hz), with HA by 4.8–10.9 dB.
  3. Moderate Flat Hearing Loss. For the digital speech signal, both APP2 and APP3 provided greater output than the HA in the low frequencies (200–400 Hz) exceeding the HA output by 9.8–13.7 dB. However, beginning at 500 Hz and extending through the mid- and high-frequency range (500–6300 Hz), both APP2 and APP3 provided less output than the HA, with HA output exceeding APP outputs by 0.5–7.6 dB.
- Comparison across hearing loss conditions for digital speech versus composite

Differences between outputs for the digital speech versus the composite signals for the three devices are shown in Table 2. Despite deactivating all the NR features in the HA, the results indicated residual NR for the steady-state composite signal, with a small effect for flat mild (up to 3.1 dB) and mild-to-moderate sloping hearing loss (up to 4.2 dB) and a moderate effect for flat moderate hearing loss (up to 5.5 dB). In contrast, APP2 and APP3 showed little to no effect across all hearing loss configurations, suggesting the absence of NR feature in AirPods device when measured in this manner.

#### 3.1.2. Condition (2) SSLP90 for Pure-Tone Sweep Signal (See Figure 2)

Across all three hearing-loss profiles, APP2 and APP3 exhibited similar SSPL90 output patterns, whereas the HA demonstrated greater profile-dependent variation, particularly in the mid-to-high frequency regions. Overall, APP3 produced higher output levels than APP2 across most frequencies (0.1 to 10.2 dB). In the low-frequency range (200–500 Hz), APP2 and APP3 showed relatively stable and comparable outputs (APP2: 86.4–92.4 dB SPL; APP3: 90–94.3 dB SPL), while the HA generally provided slightly higher output (92–96.8 dB SPL). In the mid-frequency range (800–1000 Hz), APP3 produced the highest outputs among the three devices across all hearing-loss profiles (APP2: 91.4–92.3 dB SPL; APP3: 96.5–98.4 dB SPL; HA: 80.1–85.6 dB SPL). At high frequencies (4000–6300 Hz), the HA generated the greatest output under the moderate hearing loss configuration, exceeding both APP2 (by 10.2 to 13.9 dB) and APP3 (by 5.4 to 7.5 dB). For the HA, output patterns were similar across profiles at low frequencies but diverged at higher frequencies, with the moderate flat hearing loss configuration consistently yielding greater output than the mild flat and mild-to-moderate sloping hearing loss configurations.

### 3.2. Part 2: Speech Mapping

**Figure 3:**
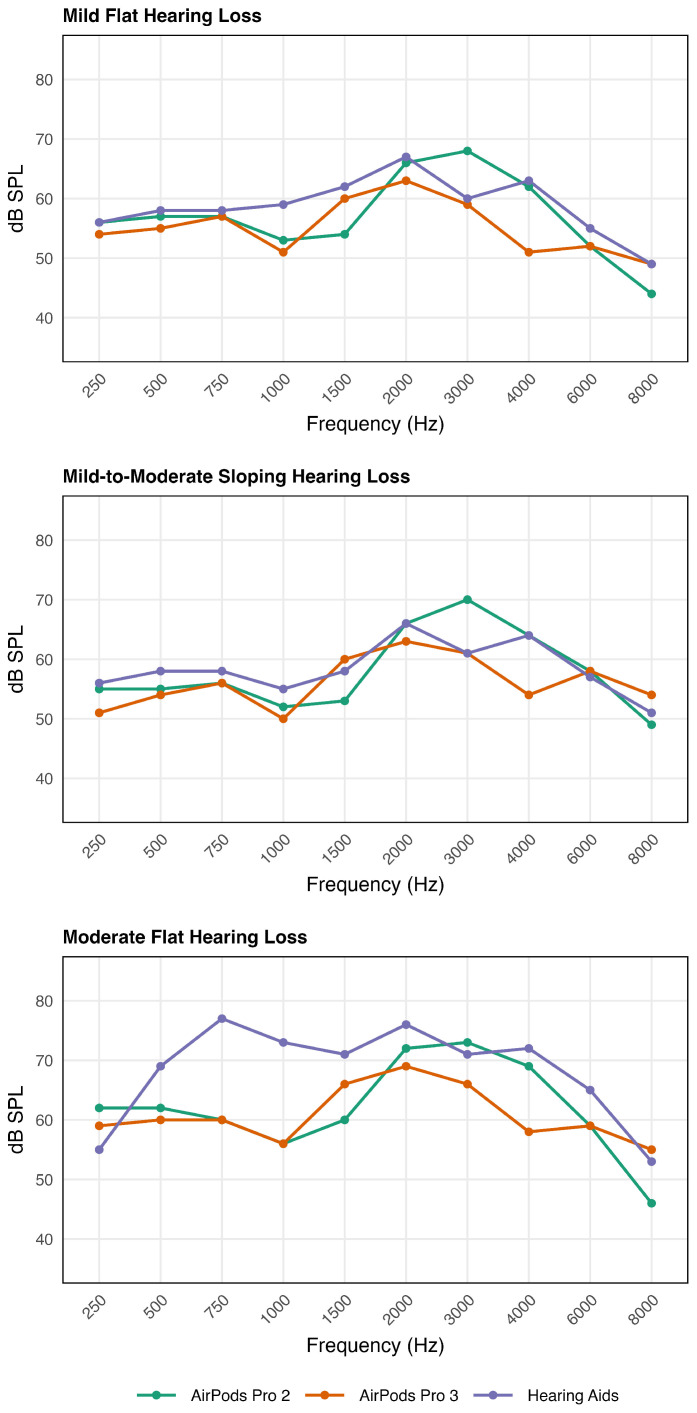
Real-ear speech mapping output for a 65 dB SPL input as a function of frequency for APP2, APP3, and HA under three hearing configurations: mild flat (**top**), moderate flat (**middle**), and mild-to-moderate (**bottom**) hearing loss (**bottom**).

**Figure 4:**
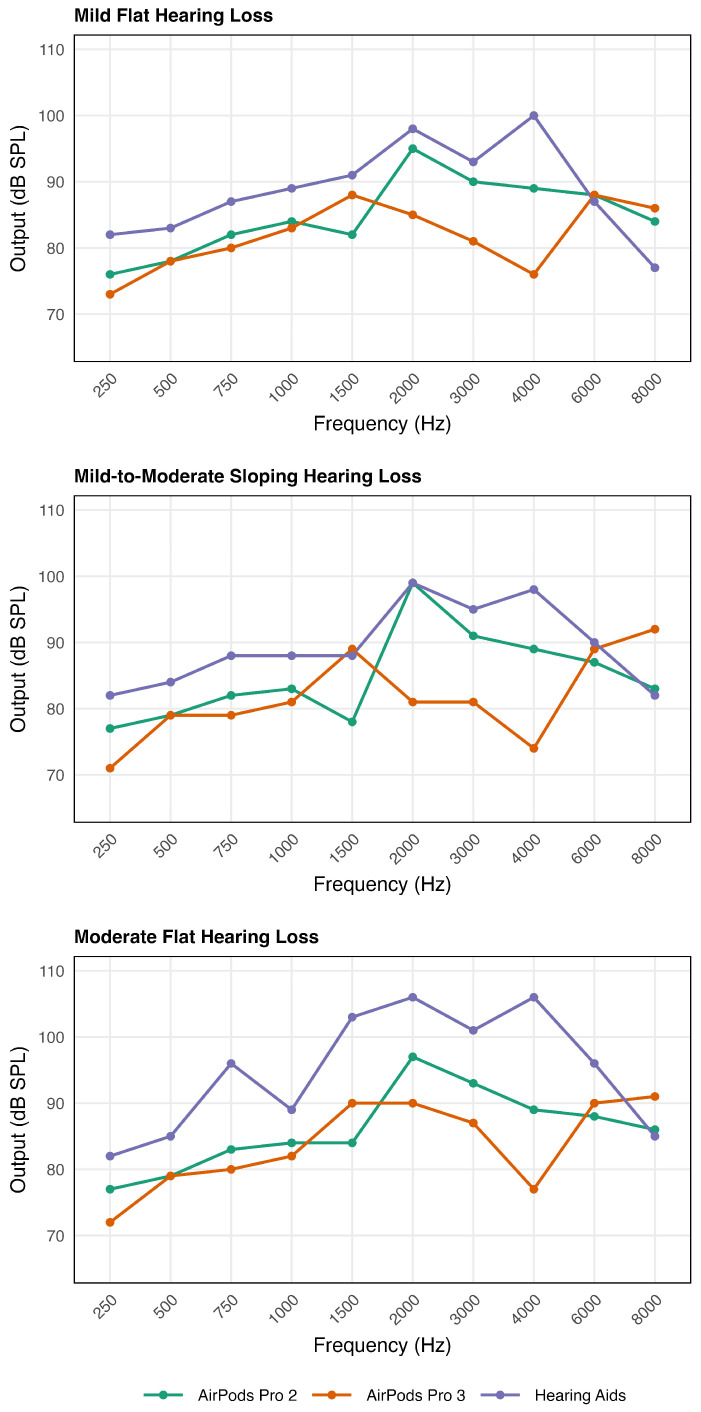
Maximum power output for an 85 dB SPL input as a function of frequency for APP2, APP3, and HA under three hearing configurations: mild flat (**top**), moderate flat (**middle**), and mild-to-moderate (**bottom**) hearing loss (**bottom**).

The results of the speech mapping are shown in Figure 3 and Figure 4. Each figure has three panels which represent the three hearing loss configurations.

#### 3.2.1. Condition (1) Speech Mapping Output Levels (See Figure 3 and Table 3)

As shown in Figure 3, moderate (2 to 17 dB) variations in real-ear output occurred across devices with especially notable deviations in the low frequencies for the moderate flat hearing loss, which are quantified in Table 3.

1. Mild Flat Hearing Loss. In the low-frequency range (250–750 Hz), APP2, APP3, and the HA showed similar amplification ranging within 0–3 dB. In the mid-frequency range (1000–2000 Hz), both APP2 and APP3 showed substantial under-amplification relative to the HA. In the high-frequency region (3000–6000 Hz), APP2 and APP3 demonstrated greater high-frequency roll-off than the HA, with APP3 showing the largest deviations. Overall, the HA generally provided highest output across all frequencies, whereas APP2 and APP3 showed slightly less amplification relative to HA, particularly above the mid-frequency range.
2. Mild-to-Moderate Sloping Hearing Loss. Across the low-to-mid-frequency range (250–2000 Hz), both APP2 and APP3 output consistently fell below that of the HA. In the high-frequency region (3000–6000 Hz), APP3 exhibited greater under-amplification relative to the HA, whereas APP2 showed amplification comparable to the HA. Overall, the HA provided the highest amplification across frequencies, whereas APP2 and APP3 showed greater deviation.
3. Moderate Flat Hearing Loss. At the lowest frequency (250 Hz), the HA produced the lowest output, whereas APP2 and APP3 produced higher output than the HA. Across the low-to mid-frequency range (500–1000 Hz), both APP2 and APP3 demonstrated substantial under-amplification relative to the HA. In the high-frequency region (2000–6000 Hz), APP2 and the HA produced comparable amplification, while APP3 demonstrated substantial under-amplification relative to the other devices. Overall, the greatest variability across devices was observed in the moderate flat hearing loss condition, with the HA providing the highest output across the low-to-high frequencies, whereas APP2 and APP3 exhibited reduced output, particularly in the low-frequency range (except for 250 Hz).

**Table 3:** Real-ear output for the HA and differences between HA and AirPods Pro by frequency and hearing loss configuration measured using Verifit 2.

		Frequency (Hz)									
		250	500	750	1k	1.5k	2k	3k	4k	6k	8k
Mild	HA output	56	58	58	59	62	67	60	63	55	49
	Diff HA-AP2	0	1	1	6	8	1	−8	1	3	5
	Diff HA-AP3	2	3	1	8	2	4	1	12	3	0
Slope	HA output	56	58	58	55	58	66	61	64	57	51
	Diff HA-AP2	1	3	2	3	5	0	−9	0	−1	2
	Diff HA-AP3	5	4	2	5	−2	3	0	10	−1	−3
Mod	HA output	55	69	77	73	71	76	71	72	65	53
	Diff HA-AP2	−7	7	17	17	11	4	−2	3	6	7
	Diff HA-AP3	−4	9	17	17	5	7	5	14	6	−2

Note. Abbreviations are the same as in Table 2 except Diff = Difference.

#### 3.2.2. Condition (2) MPO (See Figure 4)

Of all the comparisons, the greatest deviations across devices and HL conditions were observed in the MPO measurements as shown in Figure 4.

1. Mild Flat Hearing Loss. Across all frequencies, HA consistently produced the highest MPO relative to APP2 and APP3. In the low-frequency region (250–1000 Hz), APP2 provided slightly higher MPO than APP3. In the mid-frequency range (2000–4000 Hz), APP3 exhibited a pronounced reduction in MPO relative to both APP2 and the HA, yielding the lowest outputs among the three devices. At higher frequencies, MPO values converged across devices at one frequency band, followed by a reversal at the highest frequency, where both APP2 and APP3 produced greater MPO than the HA. Overall, the hearing aid demonstrated the greatest MPO across most frequencies, with frequency-dependent convergence and crossover effects observed at the highest frequencies.
2. Mild-to-Moderate Sloping Hearing Loss. Across frequencies, the HA consistently produced the highest MPO, exceeding both APP2 and APP3, with APP2 generally providing slightly greater output than APP3. In the low-frequency range (250–1000 Hz), APP2 and APP3 showed comparable outputs. In the mid frequencies (2000–4000 Hz), APP2 produced higher output than APP3, with APP3 briefly approximating HA output at 2000 Hz, whereas APP2 demonstrated a peak at 8000 Hz.
3. Moderate Flat Hearing Loss. Across all frequencies, HA consistently produced the highest MPO relative to APP2 and APP3. In the low-frequency range (250–750 Hz), APP2 and APP3 produced comparable output. In the mid-frequency range (2000–4000 Hz), APP2 provided greater output than APP3. However, in the high-frequency range (6000–8000 Hz), APP2 and APP3 again exhibited similar outputs.

### 3.3. Part 3: Real-Ear-to-Coupler Difference (See Table 4)

RECD values across devices, frequencies, and hearing loss configuration are presented in Table 4. APP2 exhibited relatively elevated RECD values in the low frequencies, followed by a gradual decline toward the high frequencies. APP3 demonstrated consistently the smallest RECD values across frequencies. In contrast, the HA showed comparatively larger RECD values in the mid-frequency range, peaking at 2000 Hz and remaining elevated through 4000 Hz. Across devices, APP2 produced greater RECD values than APP3, whereas the HA exhibited the largest RECD values overall.

**Table 4:** Real-ear-to-coupler difference values (dB) by device, frequency, and hearing loss configuration measured using Verifit 2.

		Frequency (Hz)									
		250	500	750	1k	1.5k	2k	3k	4k	6k	8k
Mild	AP2	0	2	1	2	5	15	18	14	5	−9
	AP3	1	4	2	5	13	13	11	4	5	4
	HA	5	1	10	14	14	16	13	11	5	0
Slope	AP2	5	6	6	1	3	15	19	13	6	−6
	AP3	−3	3	−1	−8	4	7	6	1	5	2
	HA	5	0	9	14	11	15	12	10	3	0
Mod	AP2	6	6	4	1	7	18	22	18	10	−8
	AP3	−1	2	−3	2	11	13	10	5	7	5
	HA	5	1	11	15	15	16	14	11	5	−1
Avg RECD		3.5	3.5	-	5.0	-	8.0	10.0	12.0	14.0	-

Note. Avg RECD = Average real-ear-to-coupler Difference (HA-1) for adults [20]; All other abbreviations are the same as in Table 2.

## 4. Discussion

The electroacoustic performance of APP2 and APP3, compared to a traditional HA, was examined across multiple domains including 2cc coupler output for continuous vs. interrupted signals, SSPL90, real-ear speech mapping, MPO, and RECD. Mild-to-moderate flat and sloping audiograms were included to reflect clinically relevant hearing loss configurations with differing frequency-specific amplification requirements. Across analyses, results demonstrated that while APP2 and APP3 are capable of providing frequency-specific amplification, their acoustic performance was limited compared to HA, particularly under the moderate hearing loss configurations.

### 4.1. Output for 65 dB SPL Input

In the mild hearing loss configuration AirPods Pro provided output comparable to the HA. However, in the mild-to-moderate sloping hearing loss configuration, insufficient amplification emerged at 4000 Hz and above, corresponding to frequencies where hearing thresholds worsened beyond 45 dB HL. Similarly, in the moderate flat hearing loss configuration, both APP2 and APP3 provided less amplification than the HA in the high-frequency region above 4000 Hz. These results suggest that although the AirPods can provide sufficient amplification in the low-to-mid frequency range, their high-frequency output is less optimal. This limitation may reduce audibility of high-frequency speech cues that are critical for speech intelligibility in noise [21,22], particularly for individuals with hearing loss greater than a moderate degree.

Interestingly, only the HA demonstrated reduced output for the composite signal relative to digital speech, while APP2 and APP3 did not show notable differences between the composite and digital speech signals. This suggests that the HA’s adaptive noise-reduction algorithms reduced output for composite stimuli, whereas the AirPods did not apply comparable noise-management adjustments when measured this way. Further study is necessary in investigating noise-management adjustment in AirPods Pro.

### 4.2. Real-Ear Measurements

Across the three hearing loss configurations, the HA consistently provided higher amplification than APP2 and APP3. Especially for moderate hearing loss condition, both APP2 and APP3 showed substantial under-amplification up to 11–20 dB below HA output from 500 to 4000 Hz, a frequency range that encompasses the fundamental frequency of the voice and supports the most benefit for speech perception [23]. Although APP2 generally provided higher amplification than APP3, it still demonstrated notable under-amplification across conditions. Together, these results indicate that while the AirPods can deliver frequency-specific gain adjustments and offer some degree of amplification, neither APP2 nor APP3 achieves prescriptive gain levels comparable to a traditional hearing aid, particularly for moderate or sloping losses, where high-frequency audibility is critical. Consequently, these limitations may negatively affect speech intelligibility and perceived sound quality for individuals with moderate HL [24].

### 4.3. SSPL90 and MPO

The SSPL90 findings indicated that APP2 and APP3 followed nearly the same output pattern across the three hearing-loss profiles, and APP3 generally reached slightly higher levels than APP2. Low-frequency outputs for both AirPods devices were relatively steady and showed minimal variation across profiles. In contrast, the HA demonstrated different patterns in SSPL90 depending on the degrees of hearing loss. Although low-frequency output was comparable across the three audiograms, the curves diverged above approximately 800 Hz, with the moderate hearing loss producing noticeably higher levels than the mild or sloping hearing loss. This pattern suggests that the HA applies hearing-loss-specific output limiting or saturation control, likely restricting maximum output for milder hearing loss configurations to avoid excessive intensity, whereas AirPods do not exhibit comparable profile-dependent output regulation.

One interesting observation was the narrow mid-frequency region around 800–1000 Hz, where APP3 briefly exceeded both APP2 and the hearing aid. Outside this region, the pattern reversed. Beginning around 1600 Hz, the HA showed a sharp increase in output for the moderate-loss condition—much higher than what was measured for either AirPods device. This trend continued into the high frequencies, where the hearing aid consistently produced the strongest saturation output. In comparison, both APP2 and APP3 rolled off more steeply, especially above 4000 Hz.

Overall, the SSPL90 patterns suggest that while the AirPods can deliver moderate maximum output in the low and mid frequencies, they do not provide sufficient output as traditional HA in the upper frequency range. This indicates that the HA is capable of delivering greater intensity and providing a larger dynamic range, making it better suited for individuals moderate hearing loss or those requiring higher output levels.

The MPO results revealed a consistent pattern across mild, moderate, and sloping hearing loss configurations, indicating that the HA produced substantially greater output than APP2 and APP3 across most frequencies. In the mild hearing loss condition, although APP2 and APP3 produced measurable MPO, APP3 showed the lowest MPO among three devices across this region. APP2 generally provided higher MPO than APP3 but remained consistently below the HA. While APP2 and APP3 exceeded the HA at 8000 Hz, this reflects the expected high-frequency roll-off of the HA rather than superior output capability of the AirPods. Differences between devices became more substantial in moderate flat hearing loss condition. The HA demonstrated markedly higher MPO across nearly all frequencies, particularly in the frequencies between 2000 and 4000 Hz. This frequency region overlaps with critical speech cues, including fricatives (e.g., s, f, sh, th), which is critical for speech clarity and distinguishing similar words [25]. In the mild-to-moderate sloping hearing loss condition, the HA again provided the most robust MPO in most of the frequencies. APP2 achieved higher MPO than APP3 across much of the mid-frequency range (1500 Hz) and APP2 briefly matched the HA at 2000 Hz; however, APP3 showed pronounced output limitations in the high frequencies, particularly around 4000 Hz, where it fell well below both APP2 and the HA.

The MPO results demonstrated substantially higher outputs for the HA compared with AirPods Pro, particularly under the moderate hearing loss condition, in contrast to the SSPL90. Whereas SSPL is measured in a 2cc coupler using steady-steady pure-tone sweeps, MPO is measured with real-ear measurement using employed pulsed (a tone burst) high-level stimuli that closely resemble speech. Differences in stimulus characteristics may have contributed to the observed discrepancy between SSPL90 and real-ear MPO measurement. MPO stimuli may have engaged device-specific compression and output-limiting mechanisms in the AirPods. Because AirPods are designed for non-clinical use, they may employ more conservative output limits to minimize the risk of over-amplification.

### 4.4. RECD

The difference between the average adult RECD (HA-1) values reported previously [20] and the measured RECD values were within 12 dB for APP2, 13 dB for APP3, and 15 dB for the HA. Overall, RECD measurements indicated the largest values for the HA, intermediate values for APP2, and the smallest value for APP3, highlighting fundamental differences in acoustic coupling and real-ear sound delivery across devices. Electroacoustic measurements are conducted in a sealed 2cc couple, which minimizes the effects of venting, leakage, insertion depth, and ear tip compliance differences, thereby reflecting the devices’ inherent output capabilities. In contrast, real-ear measurements use a probe tube inserted into the ear canal to measure the sound pressure level at the tympanic membrane while the device is worn in the ear. Lower RECD values are indicative of a poorer acoustic seal and increased leakage within the ear canal, and this pattern was observed most prominently for APP3. This discrepancy helps explain why, despite similar outputs observed during electroacoustic coupler measurements across APP2, APP3, and HA, real-ear measurements showed relatively greater amplification for the HA and APP2 and the lowest amplification for APP3. Thus, although APP3 produced higher output in the coupler, its redesigned ear tips may have provided a poorer acoustic seal in the ear canal, resulting in increased leakage and reduced in-ear amplification relative to APP2.

### 4.5. Limitations

This study was conducted using measurements from a single ear with a single ear tip size, which may not fully capture inter-subject variability, including differences in ear canal acoustics, coupling quality, and fitting conditions that can influence real-ear measurements and RECDs. Variations in ear canal geometrics, insertion depth, and seal can substantially affect acoustic coupling, potentially contributes to variability for real-ear measurements across individuals. In addition, the AirPods Pro were evaluated using only the right device and compared with a single HA model, limiting the ability to observe differences that might emerge when compared across multiple AirPods Pro units and other HA manufactured devices. Finally, this study focused solely on electroacoustic analyses and did not evaluate real-world listening outcomes. Further research should incorporate behavior and patient-centered measures, such as speech-in-noise performance, sound localization, and subjective user satisfaction, to provide a more comprehensive understanding of how these devices perform in everyday listening environments. This information is needed to fully support clinical recommendation regarding the suitability of AirPods Pro for individuals with varying degrees and configurations of hearing loss.

### 4.6. Clinical Insights

The FDA specifies that OTC HA must allow users to customize amplification to suit their individual hearing needs, including making frequency-specific adjustments. The AirPods Pro, now FDA-approved as an OTC HA, are intended for individuals with perceived mild-to-moderate hearing impairment. Although their amplification is more limited than that of prescribed HA fitted by a professional audiologist, AirPods Pro may serve as more viable alternative for individuals with mild hearing loss who seek accessible and affordable amplification and who are comfortable with navigating menus and apps on the iPhone. For those with moderate or high-frequency hearing loss, traditional prescription HA may be a more appropriate choice. Additionally, because the AirPods require users to manually input their own audiogram, it is critical to ensure accurate self-testing and proper interpretation of hearing threshold. Inaccurate audiogram entry could lead to over- or under-amplification, reduced speech audibility, and misleading perceptions of device benefit, underscoring the need for user education and clear guidance when relying on consumer-controlled health-care devices.

## 5. Conclusions

In summary, across mild, moderate flat, and mild-to-moderate hearing loss configurations, the traditional HAs consistently showed variations in output expected for the HL configuration. Although APP2 and APP3 were able to provide frequency-specific gain, both devices demonstrated reduced output relative to the HA, particularly in the low-frequency region for moderate hearing loss configurations. Overall, these findings suggest that while AirPods Pro may provide some acoustic gain for individuals with mild hearing loss or those with moderate hearing loss limited to the high-frequency region, they do not offer output that matches prescriptively fitted HAs, highlighting the continued importance of clinical verification of hearing assistive devices used in hearing rehabilitation. Further research regarding functional benefits including speech recognition and user satisfaction is needed.

## Figures and Tables

**Table 1:** Audiograms used to verify amplification across hearing loss configurations.

Audiograms (dB HL)	Frequency (Hz)							
	250	500	1k	2k	3k	4k	6k	8k
Mild flat HL	35	35	35	35	35	35	35	35
Mild-to-moderate sloping HL	25	25	30	35	40	45	50	55
Moderate flat HL	55	55	55	55	55	55	55	55

Note. HL = Hearing Loss.

**Table 2:** Output differences between digital speech and composite measured using FONIX 8000.

		Frequency (Hz)									
		200	400	500	800	1k	2k	4k	5k	6.3k	8k
Mild	AP2	−2.0	−1.5	−0.9	−0.5	−0.6	−0.8	−0.9	−1.0	−0.7	1.6
	AP3	−2.3	−0.7	0.0	−0.2	−0.2	−0.2	−0.1	−0.9	−0.5	1.2
	HA	−0.2	−0.1	0.0	0.5	2.0	2.7	2.3	2.7	2.8	1.3
Slope	AP2	−2.9	−2.2	−1.9	−1.5	−1.6	−0.5	0.4	−0.2	0.2	1.6
	AP3	−2.1	−0.9	−0.4	−0.6	−0.6	−0.2	0.5	0.1	0.1	2.4
	HA	0.5	−0.1	0.0	0.0	0.6	3.5	3.8	4.0	3.2	−1.6
Mod	AP2	−0.6	−0.2	0.5	0.5	0.3	0.1	−0.3	−0.6	0.6	1.2
	AP3	−0.3	0.0	0.4	0.6	0.2	0.1	−0.3	−0.5	−0.5	0.3
	HA	0.1	1.2	1.0	2.8	4.9	4.8	4.2	4.4	5.5	11.3

Note. AP2 = AirPods Pro 2nd generation, AP3 = AirPods Pro 3rd generation, HA = hearing aid, Mild = mild flat hearing loss, Slope = mild-to-moderate sloping hearing loss, Mod = moderate flat hearing loss.

## Data Availability

The data presented in this study are available on request from the corresponding author due to privacy considerations.

## Abbreviations

The following abbreviations are used in this manuscript:

APP2	AirPods Pro (2nd generation)
APP3	AirPods Pro (3rd generation)
HA	Hearing aid
HL	Hearing loss
RECD	Real-ear-to-coupler difference
REUR	Real-ear unaided response
REAR	Real-ear aided response
REIG	Real-ear insertion gain
MPO	Maximum power output
SSPL90	Saturation sound pressure level for a 90 dB input
SPL	Sound pressure level
SNR	Signal-to-noise ratio
NAL-NL2	National Acoustic Laboratories—Non-Linear 2 prescription
NR	Noise reduction
FDA	U.S. Food and Drug Administration
OTC	Over-the-counter
